# CXCR4^+^ Treg cells control serum IgM levels and natural IgM autoantibody production by B-1 cells in the bone marrow

**DOI:** 10.1084/jem.20220047

**Published:** 2022-06-07

**Authors:** Shlomo Elias, Rahul Sharma, Michael Schizas, Izabella Valdez, Sham Rampersaud, Sun-Mi Park, Paula Gonzalez-Figueroa, Quan-Zhen Li, Beatrice Hoyos, Alexander Y. Rudensky

**Affiliations:** 1 Immunology Program, Memorial Sloan Kettering Cancer Center, New York, NY; 2 Howard Hughes Medical Institute, Memorial Sloan Kettering Cancer Center, New York, NY; 3 Molecular Pharmacology Program, Memorial Sloan Kettering Cancer Center, New York, NY; 4 Department of Immunology and Infectious Disease, The John Curtin School of Medical Research, The Australian National University, Canberra, Australia; 5 Microarray and Immune Phenotyping Core Facility, Department of Immunology, University of Texas Southwestern Medical Center, Dallas, TX

## Abstract

Regulatory T (Treg) cells represent a specialized lineage of suppressive CD4^+^ T cells whose functionality is critically dependent on their ability to migrate to and dwell in the proximity of cells they control. Here we show that continuous expression of the chemokine receptor CXCR4 in Treg cells is required for their ability to accumulate in the bone marrow (BM). Induced CXCR4 ablation in Treg cells led to their rapid depletion and consequent increase in mature B cells, foremost the B-1 subset, observed exclusively in the BM without detectable changes in plasma cells or hematopoietic stem cells or any signs of systemic or local immune activation elsewhere. Dysregulation of BM B-1 B cells was associated with a highly specific increase in IgM autoantibodies and total serum IgM levels. Thus, Treg cells control autoreactive B-1 B cells in a CXCR4-dependent manner. These findings have significant implications for understanding the regulation of B cell autoreactivity and malignancies.

## Teaser

Inducible CXCR4 ablation in Treg cells results in dysregulation of B-1 B cells in the bone marrow due to local Treg cell depletion, leading to an increase in IgM autoantibody production and total IgM levels.

## Graphical Abstract

**Figure:**
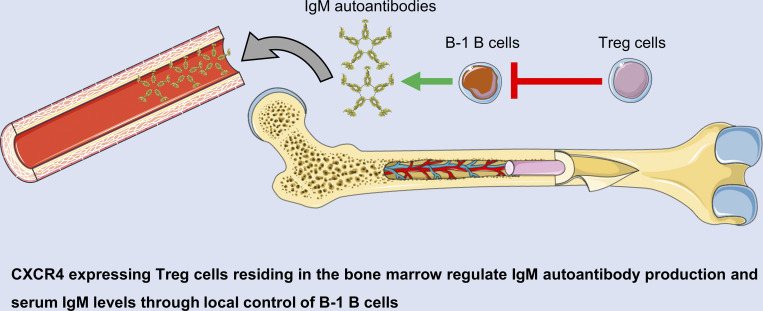

## Introduction

Cells of the immune system exhibit mixed mobile and sedentary lifestyles to confer protection of an organism against a wide range of extrinsic biotic and abiotic challenges and intrinsic perturbations of organismal homeostasis. Chemokine receptors play critical roles in enabling migration of precursors and recirculation of mature immune cells through lymphoid and nonlymphoid organs as well as in their dynamic positioning within these tissues. Shared expression of chemokine receptors with the same ligand specificity by different immune cell types facilitates their encounters. These temporally and spatially coordinated interactions are paramount for the elaboration of immune responses and their regulation. Regulatory CD4^+^ T cells (Treg cells), expressing transcription factor Foxp3, represent a specialized lineage that restrains responses of other immune cells (Fontenot et al., 2003; Hori et al., 2003; Sakaguchi et al., 1995). Immunosuppressive and tissue-supporting functions of activated Treg cells are thought to require a close opposition to their target cells with matching chemokine receptor expression. Accordingly, Treg cells express receptors for a number of proinflammatory chemokines in addition to homeostatic secondary lymphoid organ homing receptor CCR7. Indeed, restricted CCR4 deficiency in Treg cells impaired their ability to control skin and lung inflammation (Annunziato et al., 2002; Huehn et al., 2004; Iellem et al., 2003; Sather et al., 2007).

In addition to CCR7 and proinflammatory chemokine receptors, Treg cells express CXCR4, a chemokine receptor playing an important role in thymocyte differentiation and maturation, neutrophil, and B cell retention in, and release from, the bone marrow (BM; Nie et al., 2004; Suratt et al., 2004). It is noteworthy that in the thymus, in addition to guiding migration of immature thymocytes, CXCR4 signaling (in cooperation with other receptors) can promote survival of T cells. Consistent with CXCR4 function in other immune cell types, Treg cells were shown to migrate to and accumulate in the BM, where they comprise a higher proportion—up to 40–50%—of the overall CD4 T cell population than in the majority of other lymphoid and nonlymphoid tissues (Hirata et al., 2018; Zou et al., 2004). BM Treg cells exhibit increased suppressive capacity in vitro compared with their peripheral blood counterparts and display distinct gene expression features in comparison with splenic Treg cells (Camacho et al., 2020; Glatman Zaretsky et al., 2017; Zou et al., 2004). Functional studies suggested that BM Treg cells support immune-privileged status of the hematopoietic stem cell (HSC) niche, consistent with their proximity to the endosteal surface adjacent to HSCs, and that Treg cell–derived IL-10 facilitates HSCs’ supporting function of BM stromal cells (Camacho et al., 2020). BM-focused depletion of Treg cells upon selective ablation of CXCR4 or wholesale loss of Treg cells by administration of diphtheria toxin (DT) to *Foxp3*^*DTR*^ mice were suggested to increase HSC numbers and their in vitro colony-forming capacity (Hirata et al., 2018; Pierini et al., 2017). Systemic ablation of Treg cells was also reported to decrease numbers of B-lineage cells in the BM across different maturation stages including pro-B, pre-B, and mature B cells (Pierini et al., 2017). Besides proposed support for HSC maintenance and B cell differentiation, BM Treg cells, which colocalize with CD11c^+^ cells and plasma cells in the BM, are thought to support the maintenance of the latter (Glatman Zaretsky et al., 2017).

These observations suggest that expression of CXCR4 by Treg cells allows them to exert broadly targeted tissue-supporting accessory rather than immunosuppressive function in the BM, with the exception of allogeneic BM transplantation settings. This notion is confounded, however, by particularities of experimental setups in prior studies. These include generalized inflammatory responses and profound changes of organismal physiology caused by ablation of Treg cells in *Foxp3*^*DTR*^ mice, uncertain degree of CD25 antibody–mediated depletion of immature and mature cell types other than Treg cells, and potential changes in Treg cell physiology due to CXCR4 ablation in Treg cells during their thymic differentiation by a constitutively expressed Cre recombinase encoded by the *Foxp3* locus (Lee et al., 2007). Thus, the specific roles of CXCR4 expression by Treg cells and the function of CXCR4-expressing Treg cells remain poorly understood.

To gain insight into putative roles of CXCR4-expressing Treg cells in the BM, we combined their extensive characterization with tamoxifen-inducible ablation of CXCR4 in Treg cells to specifically account for their CXCR4-dependent functions. Upon temporally controlled selective depletion of Treg cells in adult BM induced by tamoxifen-triggered CXCR4 loss, we observed an increase in B-1 B cells in the BM but not in the peritoneum. These increases were associated with a marked increase in circulating IgM autoantibodies and total serum IgM levels. Thus, CXCR4-expressing Treg cells control numbers of IgM autoantibody production by B-1 B cells and overall serum IgM levels.

## Results and discussion

### Phenotypic features and stability of CXCR4-expressing Treg cells

**Figure S1.**
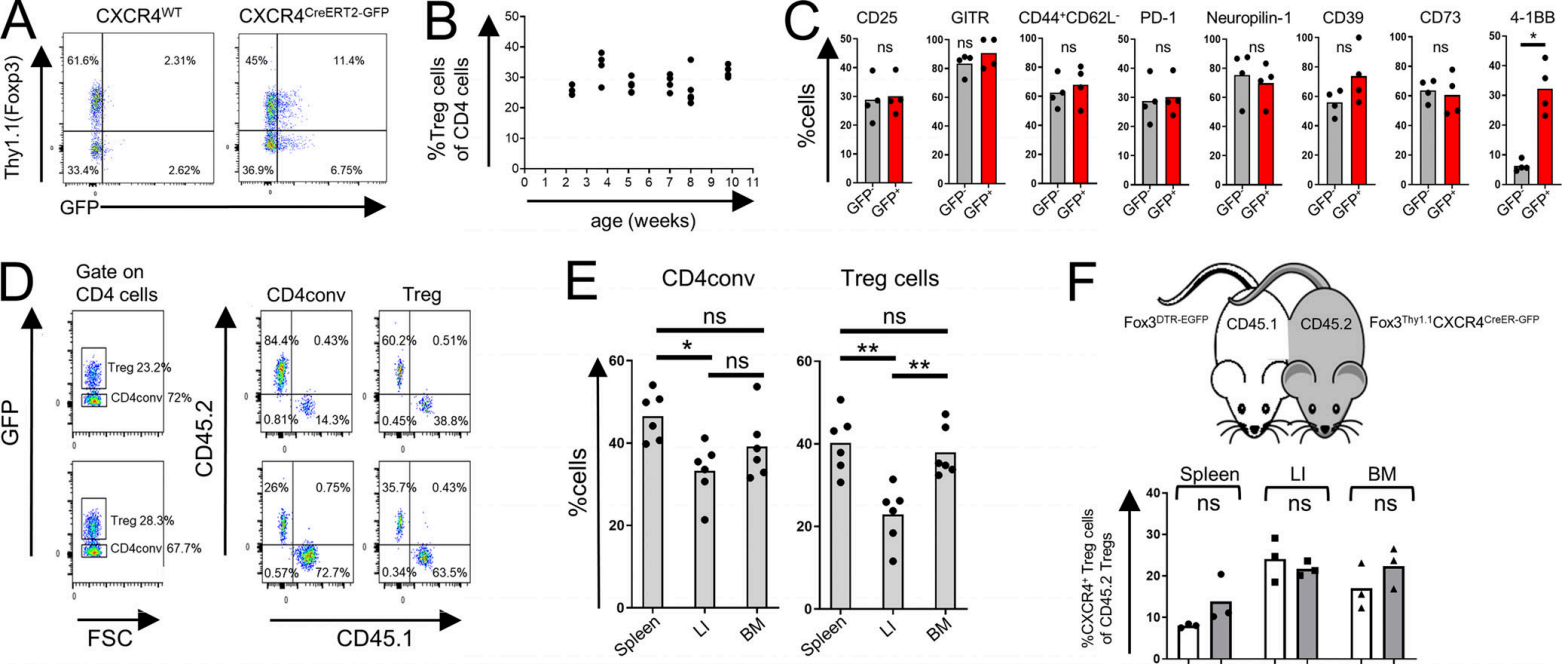
**General properties of CXCR4-expressing BM Treg cells. (A)** Flow cytometric analysis of CXCR4^+^ Treg cells in *CXCR4*^*CreERT2-GFP*^*Foxp3*^*Thy1.1*^ (right) and *CXCR4*^*WT*^*Foxp3*^*Thy1.1*^ mice (left) after gating on CD4^+^TCRβ^+^ cells. **(B)** Flow cytometric analysis of age-dependent abundance of Treg cells within CD4^+^ T cell population in *Foxp3*^DTR-EGFP^ mice. **(C)** Flow cytometric analysis of expression of activation markers by CXCR4^+^ (Thy1.1^+^GFP^+^) and CXCR4^−^ Treg cells in C*xcr4*^*CreERT2-GFP*^*Foxp3*^*Thy1.1*^ mice. The data from one experiment representative of three independent experiments are shown. *, P < 0.05, unpaired Student’s *t* test. **(D–F)** Analysis of BM Treg cells in parabiotic mice. **(D)** Gating strategies for flow cytometric analysis shown in Fig. 1 C. **(E)** Flow cytometric analysis of Treg cells in CD45.1^+^ and CD45.2^+^ *Foxp3*^*DTR-EGFP*^ parabionts 4 wk after surgery. *, P < 0.05; **, P < 0.01. ANOVA with multiple comparisons (Tukey post-hoc test). **(F)** Analysis of Treg cells in *Fox3*^DTR-EGFP^CD45.1 and *Fox3*^Thy1.1^*CXCR4*^CreERT2-GFP^CD45.2 parabionts 2 wk after surgery. The percentage of CXCR4^+^ Treg cells of CD45.2^+^ Treg cells was quantified in CD45.1^+^ (white bars) and CD45.2^+^ parabionts (gray bars). LI, large intestine. ns, not significant, unpaired Student’s *t* test.

**Figure 1.**
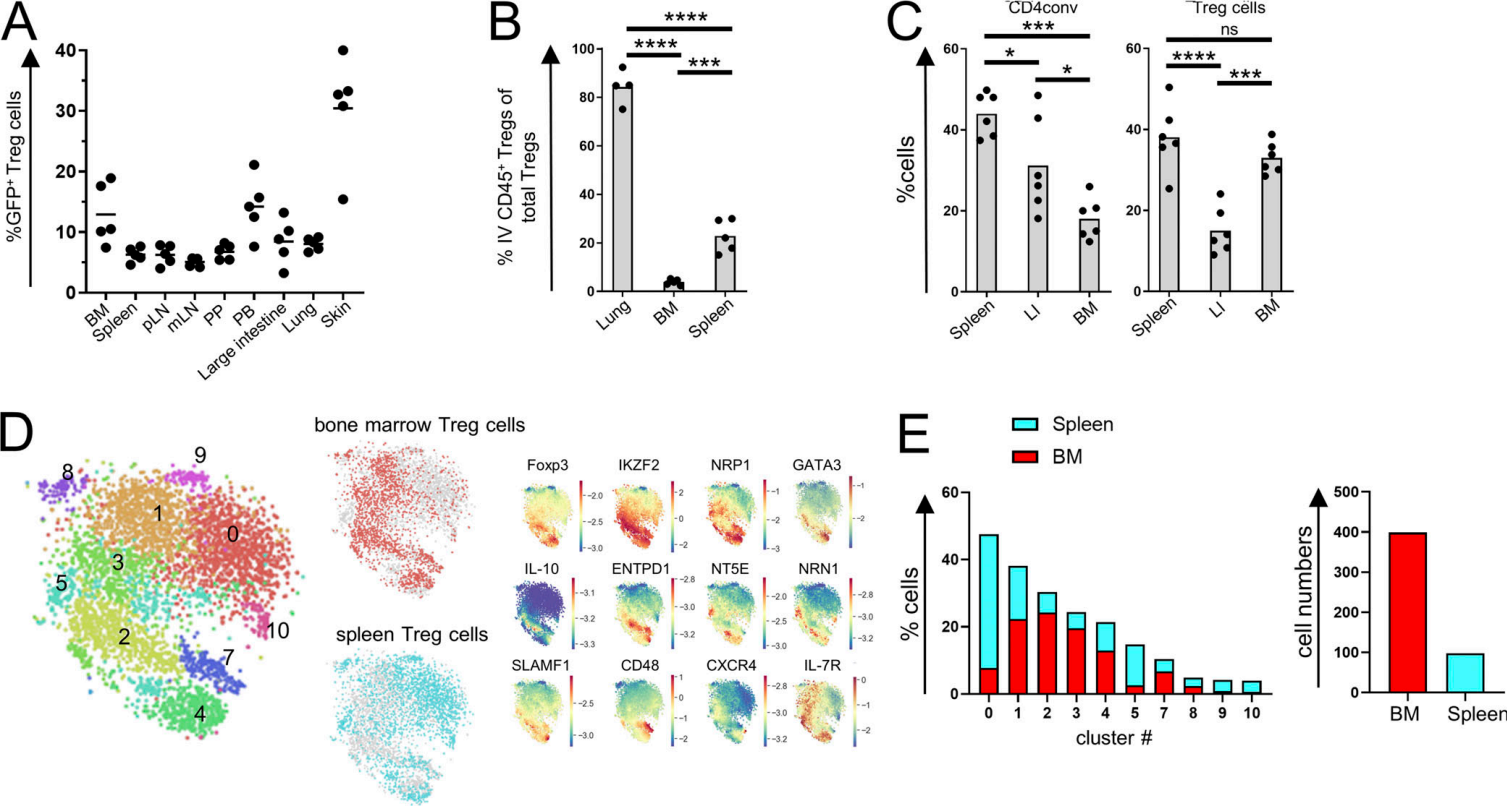
**Features of CXCR4**^**+**^ **BM Treg cells. (A)** Distribution of CXCR4^+^ Treg cells in different tissues. mLN, mesenteric lymph nodes; PB, peripheral blood; pLN, peripheral lymph nodes; PP, Peyer’s patches. **(B)** Analysis of intra- (IV) and extravascular Treg cells in *Foxp3*^*DTR-EGFP*^ mice labeled with CD45-BV570 for 3 min in the indicated tissues. **(C)** Analysis of CD4conv and Treg cells in CD45.1^+^ and CD45.2^+^ *Foxp3*^*DTR-EGFP*^ parabionts 2 wk after surgery. LI, large intestine. **(D)** t-Distributed stochastic neighbor embedding (t-SNE) visualization of scRNA-seq of spleen and BM Treg cells. Clustering analysis (left) and select gene distribution (right). **(E)** Distribution of BM and spleen Treg cells among scRNA-seq clusters (left) and CXCR4^+^ Treg numbers (right). The data from one representative experiment of two independent experiments are shown in A–C. *, P < 0.05; ***, P < 0.001; ****, P < 0.001; ANOVA with multiple comparisons (Tukey post-hoc test; B, C).

To enable quantification, tracking, and time stamping of CXCR4-expressing Treg cells, we generated *Cxcr4*^*CreERT2-GFP/WT*^*Foxp3*^*Thy1.1*^ mice, in which CXCR4^+^ Treg cells coexpressed Thy1.1 and GFP (Fig. S1 A). In agreement with previous reports (Hirata et al., 2018; Zou et al., 2004), Thy1.1^+^ Treg cells were highly enriched within total CD4 T cell population in the BM starting from 2 wk of age (Fig. S1 B), in contrast to other “Treg-rich” tissues such as skin, colon, or adipose tissue, with their markedly slower age-dependent buildup of Treg numbers. CXCR4^+^ Treg cells were most represented within BM, skin, and peripheral blood Treg populations (Fig. 1 A). We have not found significant differences in the expression level of several activation markers between CXCR4^+^ and CXCR4^−^ Treg cells, with the exception of 4-1BB, whose expression was significantly increased in CXCR4^+^ Treg cells (Fig. S1 C). Express labeling of intravascular CD45^+^ cells upon i.v. administration of a fluorophore-conjugated CD45 antibody suggested that Treg cells were residing primarily in the BM parenchyma rather than the vasculature, in contrast to other heavily vascularized organs such as lung and liver (Fig. 1 B). Nevertheless, both BM and splenic Treg cells were similarly replenished from hematogenous sources, as revealed by analysis of parabiotic CD45.1 and CD45.2 *Foxp3*^*DTR-GFP*^ mouse pairs 2 and 4 wk after surgery (33 and 38% “mixing,” respectively). This was in contrast to markedly more pronounced tissue residency exhibited by colonic Treg cells (Fig. 1 C; and Fig. S1, D and E). Analysis of the migratory activity of CXCR4^+ ^Treg cells using parabiosis of CD45.2 *Cxcr4*^*CreERT2-GFP*^*Foxp3*^*Thy1.1*^ and CD45.1 *Foxp3*^*DTR*^ mice two wk after surgery showed comparable proportion of CXCR4^+^ Treg cells among CD45.2^+^ Treg cell population in both parabionts (Fig. S1 F).

For in-depth phenotyping, we performed single-cell RNA sequencing (scRNA-seq) analysis of BM and splenic Treg cells and their conventional counterparts (CD4conv). Clustering analysis showed relative enrichment of BM Treg cells in clusters 2, 3, 4, and 7 and their underrepresentation in cluster 0 in comparison with splenic Treg cells (Fig. 1, D and E; and Table S1). Clusters 0 and 2 were enriched for resting and activated Treg cells, respectively. Cluster 2 was enriched for gene expression related to chemotaxis; clusters 4 and 7 were enriched for gene expression related to cytokine responses including IL-7 and IL-6. While overall CXCR4 transcript levels were low, they were notably increased among BM Treg cells and correlated with increased expression of *Ikzf2* (Helios), *Ifngr1*, *Il10*, *Entpd1* (CD39), *Nt5e* (CD73), *Nrn1* (neuritin), *Nrp1* (neuropilin-1), *Tnfrsf18* (GITR), *Icos*, *Hif1a*, and *Gata3* genes (Fig. 1, D and E; and Table S2). Thus, in BM Treg cells, CXCR4 expression is associated with increased expression of suppressor function–related genes.

### Induced CXCR4 ablation in Treg cells results in their selective BM depletion

**Figure 2.**
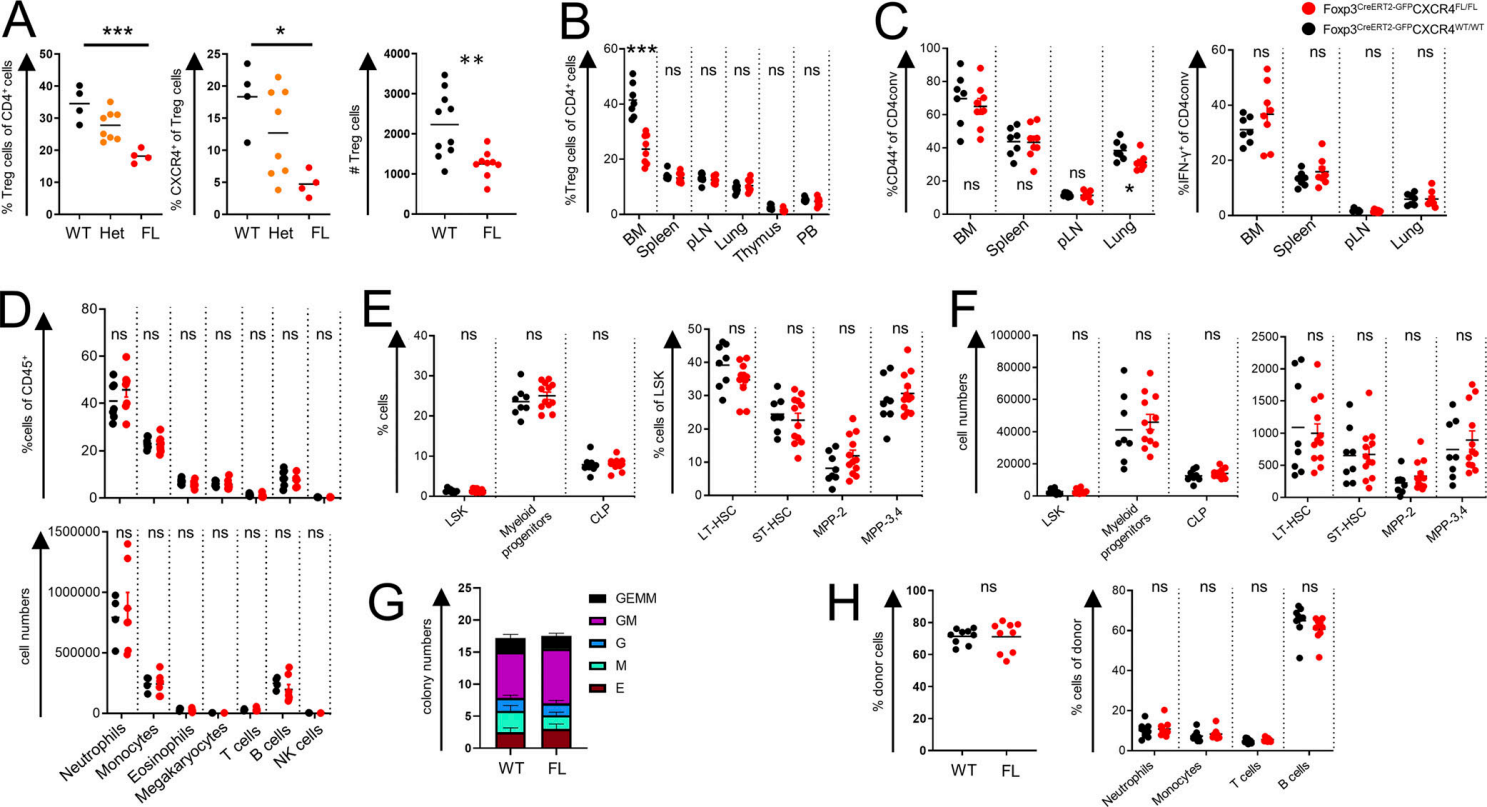
**Induced deletion of CXCR4 in Treg cells. (A and B)** Analysis of total and CXCR4^+^ Treg subsets in the BM (A) and other tissues (B) of tamoxifen-treated WT, Het, and FL mice (one representative of two independent experiments is shown). **(C)** Analysis of activated CD44^hi^CD62L^lo^ (left) and IFNγ^+^ cells (right) in tamoxifen-treated WT vs. FL mice (one representative of two independent experiments is shown). **(D–F)** Analysis of hematopoietic lineages (D), LSK cells, and other progenitor cells (E and F) in the BM of tamoxifen-treated WT and FL mice (one representative of two to four independent experiments is shown). CLP, common lymphoid progenitors; LT, long term; MPP, multipotent progenitors; ST, short term. **(G)** In vitro colony formation by BM cells from WT and FL mice (*n* = 6 of each genotype; one representative of two independent experiments is shown). **(H)** Analysis of competitive HSPC engraftment. CD45.2^+^ BM cells from tamoxifen-treated WT or FL and CD45.1^+^ BM cells were transferred at a 1:1 ratio into lethally irradiated CD45.1^+^ recipients, and peripheral blood was analyzed 12 wk later (one representative of two independent experiments is shown); *, P < 0.05; **, P < 0.01; ***, P < 0.001. ANOVA with multiple comparisons (Tukey post-hoc test; A); unpaired Student’s *t* test (A right, C, D, E, F, and H).

**Figure S2.**
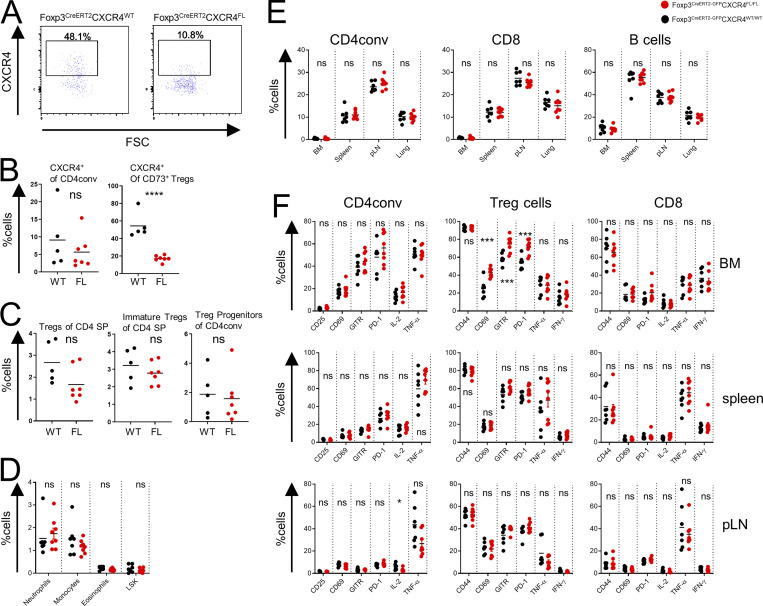
**Thymic Treg cells and activation markers in FL mice. (A–C)** Flow cytometric analysis of thymocytes in tamoxifen-treated WT and FL mice. CXCR4 expression by thymic Treg cells (A); proportion of CXCR4^+^ thymic Treg cells and CD4conv cells (B); frequencies of mature (Dump^−^CD4^+^TCRβ^+^CD8^−^CD73^−^CD25^+^Foxp3^+^) and immature (Dump^−^CD4^+^TCRβ^+^CD8^−^CD25^+^Foxp3^−^) Treg cells and Treg progenitors (Dump^−^CD4^+^TCRβ^+^CD8^−^GITR^+^CD122^+^CD25^−^Foxp3^−^) (C). The dump gate includes staining for NK1.1, CD11b, CD19, MHC-II, TCRγδ, and CD1d tetramer. **(D–F)** Flow cytometric analysis of splenocytes (D); proportion of CD4conv, CD8, and B cells subsets in indicated tissues (E); and expression of activation markers and cytokine production by CD4conv, Treg cells, and CD8 cells in different tissues (F) in tamoxifen-treated FL and WT mice. Two right columns in each panel show intracellular cytokine production after in vitro stimulation. The data are shown for one representative of two (A–C) or three (D–F) independent experiments. pLN, peripheral lymph nodes; SP, single positive. *, P < 0.05; ***, P < 0.001; ****, P < 0.0001; unpaired Student’s *t* test.

To explore the functional significance of CXCR4 expression in Treg cells, we generated *Foxp3*^*CreERT2-GFP*^*Cxcr4*^*FL/FL*^ mice, in which CXCR4 ablation in Treg cells can be induced upon tamoxifen administration, and control *Foxp3*^*CreERT2-GFP*^*Cxcr4*^*WT/WT*^ mice, hereafter termed FL and WT, respectively. Adult mice were placed on tamoxifen diet for 4–5 wk prior to analysis. Tamoxifen-induced acute ablation of CXCR4 in Treg cells resulted in their moderate but statistically significant decrease in the BM when comparing FL, heterozygous *Foxp3^CreERT2-GFP^Cxcr4^WT/FL^* (Het), and WT mice (Fig. 2 A).Therefore, FL and WT mice were used for all subsequent studies. The observed impact of the induced loss of CXCR4 on Treg numbers in the former was highly selective to the BM, as Treg cells at all other sites were unaffected (Fig. 2 B). We noted high levels of CXCR4 expression by recirculating (CD73^+^) Treg cells in the thymus; a mild decline in their numbers was merely a trend not reaching statistical significance (Fig. 2 B; and Fig. S2, A–C), and CXCR4 loss did not have noticeable effects on thymic Treg progenitors (Fig. S2 C).

The induced loss of CXCR4 in Treg cells did not result in any measurable generalized defect in their function, as tamoxifen-treated FL mice remained clinically healthy with no profound changes in CD4 and CD8 T cell activation, B cell subset composition, or proinflammatory cytokine production in the secondary lymphoid tissues and nonlymphoid organs such as lung (Fig. 2 C; Fig. S2, E and F; data not shown).

While cell surface phenotype of Treg cells was unchanged, we noted an increase in several activation markers (CD69, GITR, and PD-1) limited to the BM Treg subset, a likely reflection of compensatory activation caused by their diminished numbers (Fig. S2 F).

Next, we sought to assess whether the loss of CXCR4 expression by Treg cells and their consequent decrease in the BM affected generation and number of innate immune cell lineages. We found major leukocyte subsets to be numerically unchanged in the BM of tamoxifen-treated FL mice (Fig. 2 D), with no detectable extramedullary hematopoiesis in the spleen (Fig. S2 D). Contrary to a previous suggestion that BM Treg cells control turnover and numbers of HSCs in a CXCR4-dependent manner (Hirata et al., 2018), we found that Lin^−^Sca1^+^cKit^+^ (LSK) cells and their subsets, including long-term HSCs defined as CD150^+^CD48^−^ LSK cells, were unaffected by the induced Treg-restricted CXCR4 deficiency (Fig. 2, E and F). We also did not notice differences in common lymphoid (Lin^−^CD127^+^FLT-3^+^) or myeloid (Lin^−^Sca1^−^cKit^+^) progenitor subsets (Fig. 2, E and F). Furthermore, in vitro colony-forming activity of HSCs isolated from tamoxifen-treated WT and FL mice (Fig. 2 G) and their potential for competitive reconstitution of lethally irradiated hosts, when admixed with CD45.1^+^ BM cells at a 1:1 ratio, were comparable (Fig. 2 H).

### CXCR4 loss by Treg cells selectively affects the BM B cell compartment

**Figure 3.**
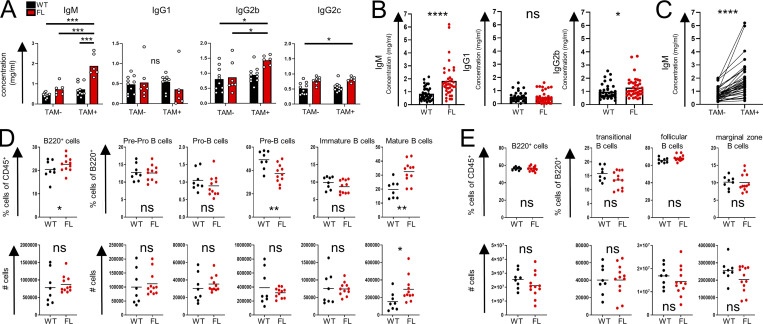
**CXCR4 deletion in Treg cells results in an increase in serum IgM and mature BM B cells. (A)** Serum Ig isotype levels of WT and FL mice before and after tamoxifen-induced CXCR4 deletion (one representative experiment of four experiments). **(B and C)** Aggregate changes in IgM, IgG1, and IgG2b levels in WT vs. FL mice after tamoxifen treatment (B) as well as aggregate changes in IgM before and after tamoxifen administration in FL mice (C); (four independent experiments). **(D)** BM B cell subsets shown as fraction of CD45^+^ or B220^+^ cells (upper panels) and absolute numbers (lower panels; one representative experiment of five). **(E)** Splenic B cell subsets in WT and FL mice (one representative experiment of three). *, P < 0.05; **, P < 0.01; ***, P < 0.001; ****, P < 0.0001. ANOVA with multiple comparisons (Tukey post hoc analysis) (A); paired Student’s *t* test (C); unpaired Student’s *t* test (B, D, and E).

**Figure S3.**
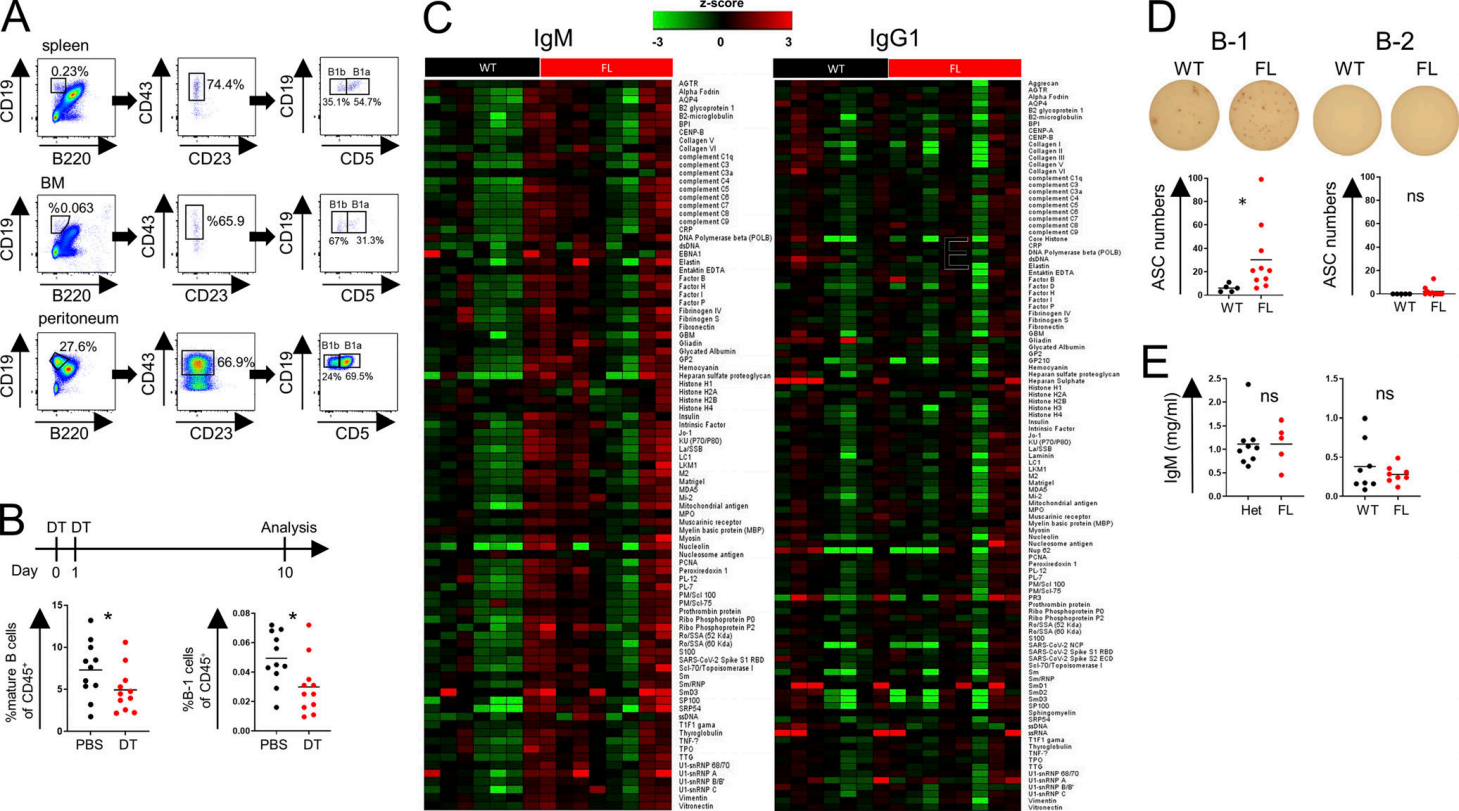
**BM Treg cells regulate B-1 cells. (A)** B-1 cells gating strategy. **(B)** Staining of mature B cells (left) and B-1 cells (right) in the BM in Fox3^DTR^ mice 10 d after two DT or PBS (control) injections. Data are combined from two independent experiments with at least five mice per group. **(C)** Heatmap of IgM and IgG1 autoantibodies. **(D)** ELISPOT analysis of BM B cells. BM B-1 (left) and B-2 cells (right) were sorted after CD19 magnetic bead enrichment and plated on ELISPOT plates precoated with five autoantigens: core histone, ribo phosphoprotein P2, intrinsic factor, mitochondrial antibody, and SP100. **(E)** IgM levels in *Foxp3*^*CreERT2*^*IL-10*^*FL/FL*^ after treatment with tamoxifen (left) and *Foxp3*^*Cre*^*Nrn1*^*FL/FL*^ (right). *, P < 0.05; unpaired Student’s *t* test (B, D, and E).

Upon further characterization of the immune status of mice harboring CXCR4-deficient Treg cells, we observed a notable and highly selective increase in serum IgM, while amounts of other Ig isotypes including IgG1, IgG2b, and IgG2c were comparable or only mildly increased in tamoxifen-treated FL vs. WT mice (Fig. 3 A). Consistently, combined analysis of serum IgM levels in WT and FL mice after tamoxifen treatment (Fig. 3 B, left) and serum IgM levels in FL mice before and after tamoxifen (Fig. 3 C) from different experimental cohorts showed a reproducible rise in IgM levels in FL mice. Similar analysis showed unsignificant changes in IgG1 and negligible, even if statistically significant, elevation in IgG2b (Fig. 3 B, right). To test whether the changes in IgM levels reflected numerical changes in a specific B cell subset despite largely unaltered bulk peripheral and BM B cell population sizes, we performed a finer-grain flow cytometric analysis of immature and mature B cell subsets in WT and FL mice (gated on live CD45^+^GR-1^−^TCRβ^−^B220^+^ cells): pre-pro-B cells (CD43^+^CD24^−^BP-1^−^), pro-B cells (CD43^+^CD24^+^BP-1^−^/CD43^+^CD24^+^BP-1^+^), pre-B cells (CD43^−^IgM^−^IgD^−^), immature B cells (CD43^−^IgM^+^IgD^−^), and mature B cells (CD43^−^IgM^+^IgD^+^). We observed a significant reduction in the proportion, but not the numbers, of pre-B cells and an increase in both proportion and absolute numbers of mature B cells in the BM (Fig. 3 D). In contrast, tamoxifen-treated FL and WT mice harbored comparable splenic B cell subsets including transitional (B220^+^CD93^+^), follicular (B220^+^CD93^−^CD19^+^IgM^low^CD21^−/low^), and marginal zone (B220^+^CD93^−^CD19^+^IgM^+^CD21^+^) B cells and total B cell populations in the lymph nodes, spleen, and lung as noted above (Figs. S2 E and 3 E). Thus, deletion of CXCR4 in Treg cells led to an increase of mature B cells in the BM and in serum IgM levels, whereas other isotypes were unaffected or only marginally affected.

### CXCR4-expressing Treg cells control B-1 cells in the BM

**Figure 4.**
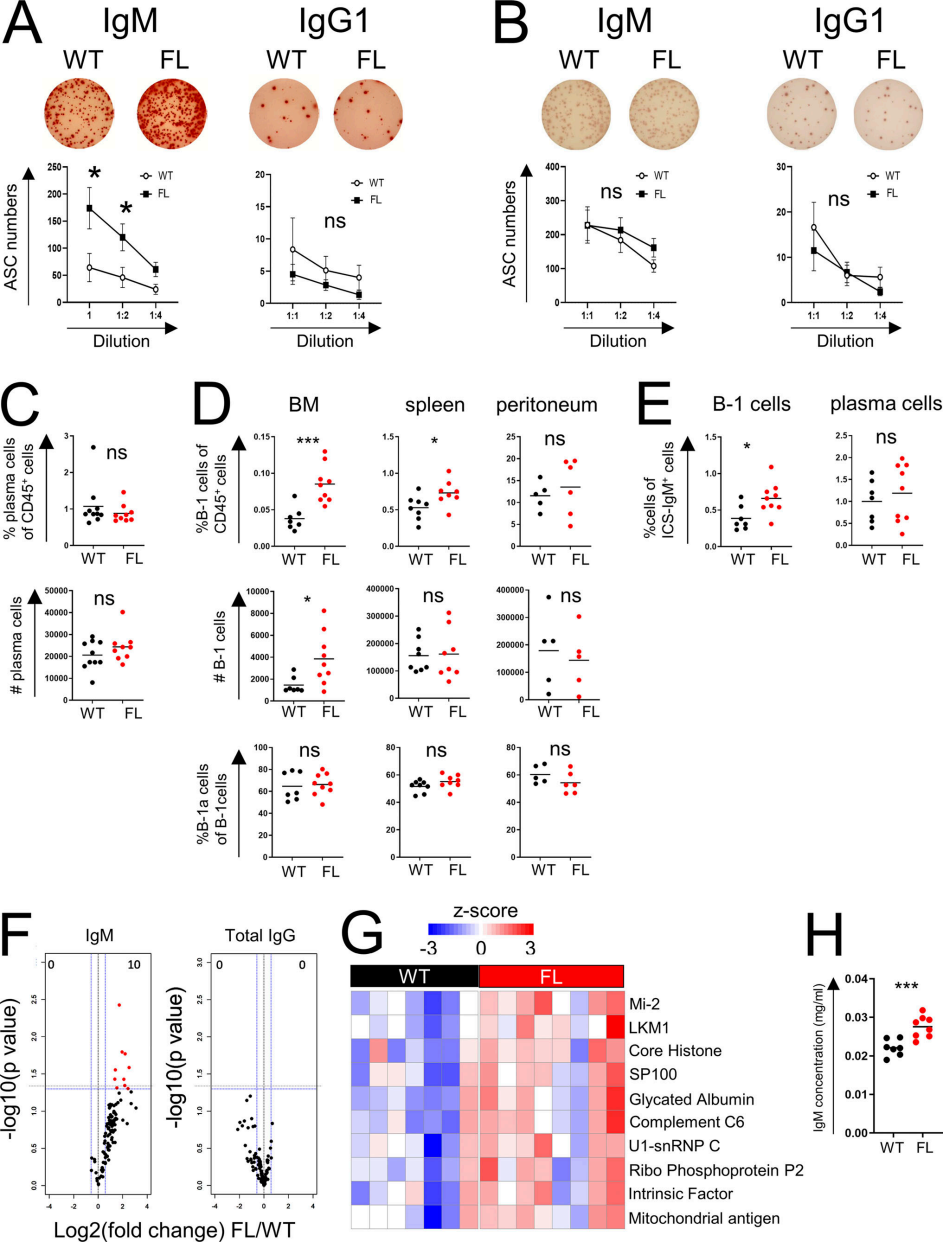
**BM Treg cells control B-1 cells. (A and B)** ELISPOT analysis of BM (A) and spleen cells (B) of tamoxifen-treated WT and FL mice. **(C and D)** Quantification of plasma cells in the BM (C) and B-1 cells in the indicated tissues (D) in tamoxifen-treated WT and FL mice. **(E)** Flow cytometric analysis of intracellular IgM expression. The percentages of intracellular IgM^+^ cells within indicated cell subsets are shown. **(F and G)** Analysis of reactivity of serum antibodies in tamoxifen-treated WT and FL mice with a 128-autoantigen microarray. Volcano plots (F; left, IgM; right, IgG; red, highly ranked) and heatmap (G) of the mean intensity of autoantibody reactivity in FL vs. WT mice. **(H)** Validation by ELISA of the concentration of the most significant IgM autoantibodies. For A–E, one representative experiment is shown of at least two. *, P < 0.05; ***, P < 0.001; unpaired Student’s *t* test.

To identify the potential source of the increased IgM amounts in FL mice, we performed ELISPOT assays of BM and spleen cells (Fig. 4 A). IgG1 served as a negative control in these experiments because its levels were similar in FL and WT mice (Fig. 3 A). We noted a significant and highly selective increase in IgM-producing, but not IgG1-producing, antibody-secreting cells (ASCs) in FL vs. WT BM (Fig. 4 A), while the frequencies of both IgM- and IgG1-producing ASCs in the spleen were comparable in WT and FL mice (Fig. 4 B). These observations suggested that the higher levels of serum IgM we observed in FL mice resulted from increased ASCs in the BM of FL compared with WT mice.

Because plasma cells and B-1 B cells are thought to serve as major IgM-producing cell types in unchallenged mice, we assessed their frequencies in WT and FL mice (Fig. 4 C). Although we observed comparable numbers of CD138^+^B220^−^ plasma cells (Fig. 4 C), we found markedly higher proportions and numbers of B220^−^CD19^+^CD23^−^CD43^+^ B-1 cells in the BM, but at best a marginal increase in the spleen, and no change in the peritoneal cavity (Figs. 4 D and S3 A). Consistent with the possibility of B-1 cells contributing to heightened serum IgM levels, the proportion of B-1 cells, but not plasma cells expressing intracellular IgM, was also increased in tamoxifen-treated FL vs. WT mice (Fig. 4 E). We did not observe increases in BM B-1 cells or mature B-2 cells upon transient depletion of Treg cells in *Foxp3*^*DTR*^ mice (Fig. S3 B).

Considering a known bias of B-1 cell specificity toward “self,” we analyzed reactivity of circulating IgM and total IgG antibodies in these mice using a panel of 128 known autoantigens. Although the titers of total IgG autoantibodies were comparable in tamoxifen-treated FL vs. WT mice, in the former we observed increased amounts of IgM autoantibodies directed against a range of autoantigens. Most prominent among them were Mi-2, LKM1, core histone, and SP100 (Fig. 4, F and G; and Fig. S3 C). These results were confirmed by ELISA using 10 representative autoantigens from this panel (Fig. 4 H). Furthermore, ELISPOT assay revealed increased frequency of BM B-1 cells, producing autoantibody against a pool of these predominant autoantigens, in FL vs. WT mice, whereas no significant autoantibody production by BM B-2 cells was observed (Fig. S3 D).

These results suggest that under physiologic conditions, BM Treg cells regulate, in a CXCR4 dependent manner, the levels of self-reactive serum IgM through control of their production by B-1 B cells in the BM. Although the identity of a Treg-derived factor or factors acting directly or indirectly upon B-1 cells remains unknown, we observed that the two major “suspects”—neuritin recently implicated in control of GC B cells by follicular Treg cells (Gonzalez-Figueroa et al., 2021) and Treg-derived IL-10—are fully dispensable for Treg-mediated control of serum IgM levels (Fig. S3 E).

Besides exerting their widely recognized generic suppressor activity, Treg cells residing in lymphoid and nonlymphoid tissues also contribute to certain specific aspects of tissue functions in physiological and pathological settings (Munoz-Rojas and Mathis, 2021; Panduro et al., 2016). These specialized organ-specific “homeostatic” roles of Treg cells remain poorly understood, in part because the majority of studies have relied on wholesale depletion or constitutive genetic perturbations of Treg cells. Pronounced systemic autoimmunity and inflammation caused by the former can obscure more nuanced functions of Treg cells, while the latter may affect the “baseline” state of developing tissue. Here, we show that temporally controlled interference with the migration of Treg cells to, and their residence within, a given tissue can alleviate these confounders and reveal novel, previously unappreciated facets of Treg biology. Through inducible ablation of the chemokine receptor CXCR4 in Treg cells causing their partial and highly selective depletion in the BM, we uncovered a novel role of CXCR4-expressing BM Treg cells in controlling B-1 cell numbers and autoantigen-specific IgM production by these cells exclusively in the BM. This is consistent with the notion that BM B-1 cells serve as major IgM producers and main contributors to the circulating IgM pool in physiologic conditions (Choi et al., 2012; Reynolds et al., 2015; Savage et al., 2017). In agreement with the aforementioned notion of “masking” effects of systemic Treg depletion, the latter failed to increase BM B-1 or mature B-2 cell numbers.

Importantly, the observed effects of CXCR4 were not associated with any noticeable changes in immune balance outside the BM, in numbers of HSCs or their colony-forming and BM reconstitution capacity. These findings differ from previous studies suggesting that a sizeable proportion of BM Treg cells are found in the proximity of the endosteal surface—a site of primitive HSC niches—and implicating IL-10 and adenosine production by BM Treg cells in HSC maintenance (Fujisaki et al., 2011; Hirata et al., 2018). Depletion of Treg cells induced by DT in *Foxp3*^*DTR*^ mice or by depleting CD25 antibodies has been associated with a reduction in the numbers of allo-HSCs and hematopoietic stem and progenitor cells (HSPCs; Fujisaki et al., 2011). Furthermore, constitutive deletion of a *Cxcr4* conditional allele in Treg cells was shown to increase BM cellularity and HSPC and HSC numbers and enhance BM reconstitution upon BM transplantation (Hirata et al., 2018). Beyond the aforementioned potential effects of pronounced inflammatory responses induced upon Treg ablation on HSCs, additional explanation for the apparent discrepancy between these previous findings and our observation that CXCR4-expressing Treg cells were dispensable for HSC maintenance is potential developmental alterations in the BM of mice harboring CXCR4-deficient Treg cells. In support of this possibility, we observed pronounced enrichment of Treg cells among overall CD4 T cell population in the BM as early as 2 wk of life as opposed to their much slower build-up in other “Treg-rich” organs.

While the sparsity of both B-1 cells and Treg cells in the BM presents a major obstacle to a rigorous assessment of their relative proximity, we propose that BM Treg and B-1 cells localize to the same niche guided by the their shared expression of CXCR4, with CXCL12-expressing stromal cells serving as their meeting points in the BM. CXCR4 is known to be expressed by all subsets of B cells through B cell ontogeny, and mice deficient in CXCR4 or CXCL12 display defects in B cell development (Epling-Burnette et al., 2007; Tachibana et al., 1998; Zou et al., 1998). Importantly, selective loss of CXCR4 in B cells results in premature migration of B cell precursors from the BM and their localization in splenic follicles (Nie et al., 2004). It has also been shown that the homing of mature B cells to the BM depends on CXCR4 expression (Beck et al., 2014; Nie et al., 2004; Pereira et al., 2009). Although these findings provide rationale for direct interactions between BM Treg and B cells, their indirect interactions through stromal cells, including osteoblasts, are also possible, considering a role for stromal cells in B cell development (Wu et al., 2008). Moreover, such interactions with stromal cells could affect the level of IL-7, which is important for both B-2 and B-1 cell development (Dias et al., 2005; Esplin et al., 2009).

Despite considerable research efforts, the control mechanism of B-1 B cells and natural IgM levels remained unknown (Baumgarth, 2017). Our studies demonstrate that Treg cells, in a CXCR4 dependent manner, control IgM autoantibody production by B-1 B cells, highlighting a novel mechanism of cell-extrinsic regulation of BM B-1 B cells and physiologic amounts of serum IgM and their self-reactivity. These results may have implications for understanding the pathogenesis of autoimmune and B cell lymphoproliferative diseases such as chronic lymphoproliferative leukemia, which has been linked to a human counterpart of mouse B-1 B cells (Hayakawa et al., 2016; Herve et al., 2005).

## Materials and methods

### Mice

*Foxp3*^*CreERT2*^, *Foxp3*^*Thy1.1*^, *Foxp3*^*DTR*^, and *CXCR4*^*CreERT2*^ mice were described elsewhere (Kim et al., 2007; Liston et al., 2008; Rubtsov et al., 2010; Werner et al., 2020). *CXCR4*^*FL*^, B6 CD45.1 mice were purchased from The Jackson Laboratory. The experimental mice on C57BL/6J (B6) background were maintained in the specific pathogen–free animal facility at Memorial Sloan Kettering Cancer Center. All animal experiments were approved by the institutional animal care and use committee of Memorial Sloan Kettering Cancer Center (08-10-023). 5–12-wk-old mice were used for all experiments unless mentioned otherwise and were age- and sex-matched in individual experiments. For induced CXCR4 deletion in Treg cells, mice were placed on a tamoxifen citrate–containing diet (TD.130860; Envigo) for 4–5 wk. For DT-induced Treg ablation, *Foxp3*^DTR^ mice received i.p. injection of 500 ng DT (List Labs) on days 0 and 1, and tissues were analyzed on day 10.

### Parabiosis and i.v. labeling

Female CD45.1 and CD45.2 B6 mice underwent surgery to establish parabiosis, as previously described (Fan et al., 2018; Kamran et al., 2013). For i.v. labeling of CD45^+^ cells, mice received i.v. injection of 3 μg of a Brilliant Violet 570–conjugated CD45 antibody (BioLegend) and sacrificed 3 min later, and the fraction of i.v.-labeled cells was quantified.

### Tissue preparation and cell isolation

Spleens, lymph nodes, and thymi were mechanically dissociated with the back of a syringe plunger and filtered through a 100-µm cell strainer (Corning). For isolation of colon lamina propria cells, the colon was first incubated with EDTA solution containing PBS, 2% FBS, 10 mM Hepes buffer, 1% penicillin–streptomycin, 1% L-glutamine, 1 mM EDTA, and 1 mM dithiothreitol at 37°C for 15 min while shaking. After centrifugation, the intraepithelial fraction was removed. Colon tissue samples devoid of intraepithelial cells and lung and skin samples were enzymatically digested with 1 mg/ml Collagenase A from *Clostridium histolyticum* (Roche) and 1 U/ml DNaseI (Sigma-Aldrich) with 2% FBS, 1% penicillin–streptomycin, 1% L-glutamine, and 10 mM Hepes in the presence of 0.25-inch ceramic spheres (MP Biomedicals) for 45 min at 37°C while shaking. Digested tissue was filtered through 100-µm cell strainers, and colon and lung cells were further enriched by 40% Percoll (GE Healthcare) centrifugation. For isolation of BM cells, one tibia and one femur from each mouse were cut at their ends and placed into a 0.2-ml microcentrifuge tube that was previously pierced by an 18G needle (Amend et al., 2016). The microcentrifuge tube was placed into a 1.5-ml microcentrifuge tube and centrifuged at 2,000 *g* for 1 min. Spleen, BM, lung, and liver cell suspensions were subjected to red blood cell lysis before downstream applications.

### Flow cytometric analysis and cell sorting

Single-cell suspensions were resuspended in PBS and stained with Ghost Dye Violet 510 (Tonbo Biosciences) for dead-cell exclusion in the presence of anti-CD16/32 (Tonbo Biosciences) to block binding to Fc receptors for 10 min at 4°C. Cell surface antigens were stained for 20 min at 4°C in FACS buffer (PBS, 2% BSA, 2 mM EDTA, 1% L-glutamine, and 10 mM Hepes). Cells were analyzed unfixed or fixed with 1% paraformaldehyde (Electron Microscopy Sciences) for later analysis or fixed and permeabilized with the BD Cytofix/Cytoperm kit for intracellular staining. Fixation, permeabilization, and intracellular staining were performed as recommended by the manufacturer. All cells were resuspended in FACS buffer and filtered through 100-µm nylon mesh before analysis on the flow cytometer. To aid acquisition, samples were treated with 40 U/ml DNase I for 10 min at 37°C before acquisition. 123count eBeads (eBioscience) were added at 5,000 beads per sample to quantify absolute cell numbers. All flow cytometry data were collected on a BD LSRII (BD) flow cytometer or the Aurora spectral analyzer (Cytek) and analyzed using FlowJo 10 software (TreeStar). Cell suspensions were stained with fluorescence-tagged antibodies. To assess cytokine production after ex vivo restimulation, single-cell suspensions were incubated in round-bottom 96-well plates for 3 h at 37°C with 5% CO_2_ in the presence of 50 ng/ml PMA and 500 ng/ml ionomycin with 1 µg/ml brefeldin A (all Sigma-Aldrich) and 2 µM monensin (BioLegend). For intracellular staining of IgM, cells were first incubated with an unconjugated IgM antibody (10 μg/ml; clone II/41; eBioscience) together with fluorophore-tagged panel of surface marker–specific antibodies for 30 min on ice. Then the cells were fixed and permeabilized (BD Cytofix/Cytoperm kit) and stained intracellularly with PE-conjugated anti-IgM antibody (clone II/41; eBioscience).

### Serum Ig ELISA

ELISA was performed for quantification of the concentration of total serum Ig and autoantibodies. For ELISA experiments, mouse peripheral blood collected by retroorbital bleeding under isoflurane anesthesia or inferior vena cava blood collected by terminal bleeding was collected into BD SST microcontainer tubes, and sera were harvested after centrifugation. Nunc-Immuno MaxiSorp F-bottom 96-well plates were coated with 50 μl/well capture antibodies in PBS, pH 7.4, overnight at 4°C. The plates were then washed three times with PBS containing 0.05% Tween-20 (Sigma-Aldrich), blocked with 150 μl ELISA/ELISPOT Diluent (Life Technologies Corp.) for 3 h, and washed with PBS containing 0.05% Tween-20. 50 μl of sera at appropriate dilutions was added and incubated overnight at 4°C. The plates were then washed with 0.05% PBS–Tween-20 and incubated with 50 μl of goat anti-mouse Ig-HRP at room temperature for 2 h, washed seven times with 0.05% PBS–Tween-20, and developed with 50 μl of tetramethylbenzidine solution (5120-0047; SeraCare) at room temperature. The colorimetric reaction was stopped with 50 μl of 1 M H_3_PO_4_ (P5811; Sigma-Aldrich) after 1–5 min of adding tetramethylbenzidine and absorbance at 450 nm was measured with a Synergy HTX plate reader (BioTek). Concentrations of antigens were determined using standard curves constructed with purified recombinant proteins and calculated with Gen5 3.02.2 (BioTek).

For determining the concentration of total serum immunoglobulins, we used the following capture antigens at 2 μg/ml in PBS: goat anti-mouse IgM (RRID: AB_2794197, 1020-01; SouthernBiotech), goat anti-mouse IgG1 (RRID: AB_2794408, 1070-01; SouthernBiotech), goat anti-mouse IgG2b (RRID: AB_2794517, 1090-01; SouthernBiotech), and goat anti-mouse IgG2c (RRID: AB_2794464, 1079-01; SouthernBiotech). We used HRP conjugated goat anti-mouse Ig (RRID: AB_2728714, 1010-05; SouthernBiotech) 1:5,000 for detection.

For IgM autoantibodies, ELISA plates were coated with the following autoantigens (3 μg/ml in PBS each): Mi-2 (A18100; Surmodics), LKM 1 (A31800; Surmodics), core histone (11010; Cayman Chemical Co.), SP100 (A18900; Surmodics), glycated albumin (A8301; Sigma-Aldrich), complement C6 (A404; Quidel), U1-snRNP C (A13200; Surmodics), ribophosphoprotein P2 (A14300; Surmodics), intrinsic factor (A16701; Surmodics), and mitochondrial Ag (ATM02-10; Arotec Diagnostics). IgM autoantibody was visualized by HRP-conjugated anti-mouse IgM (1:5,000, 1020-05; SouthernBiotech).

We used the following reagents to construct standard curves for ELISA in this study: IgM isotype control from murine myeloma (M5909; Sigma-Aldrich); purified mouse IgG1, κ, isotype control (0102-01; SouthernBiotech); IgG2b isotype control from murine myeloma (M5534; Sigma-Aldrich); and mouse IgG2c (RRID: AB_2794064, 0122-01; SouthernBiotech).

### Measurement of serum autoantibodies using autoantigen array

Serum autoantibodies were analyzed using an autoantigen array chip containing 128 antigens and controls at Microarray and Immune Phenotyping Core Facility (University of Texas Southwestern Medical Center). Briefly, the autoantigens and control proteins were printed in duplicate onto nitrocellulose film slides (Grace Bio-Labs). 2-μl serum samples were pretreated with DNAse-I and then diluted 1:50 in PBS-Tween buffer for autoantibody profiling. The diluted serum samples were incubated with the autoantigen arrays, and autoantibodies binding with antigens on arrays were measured with cy3-conjugated anti-human IgG (1:2,000; Jackson ImmunoResearch Laboratories) and cy5-conjugated anti-human IgM (1:1,000; Jackson ImmunoResearch Laboratories), using a Genepix 4200A scanner (Molecular Device) with laser wavelength of 532 and 635 nm. The resulting images were analyzed with Genepix Pro 7.0 software (Molecular Devices). The median of the signal intensity for each spot was calculated and subtracted from the local background around the spot, and data obtained from duplicated spots were averaged. The background-subtracted signal intensity of each antigen was normalized to the average intensity of the human IgG or IgM controls, which were spotted on the array as internal controls. Finally, the normalized fluorescence intensity was generated as a quantitative measurement of the binding capacity of each antibody with the corresponding autoantigen. Signal-to-noise ratio (SNR) is another quantitative measurement of the true signal above background noise. SNR values ≥3 were considered significantly higher than background, and therefore true signals. The autoantibody with an SNR value <3 in >90% of samples was considered negative and excluded from further analysis. The normalized fluorescence intensity value of the autoantibodies was used for statistical analysis, and heatmaps were generated using Cluster and Treeview software (http://bonsai.hgc.jp/∼mdehoon/software/cluster/software.htm). Statistical analysis was performed using R package to identify differential expressed antibodies between different study groups.

### ELISPOT

ELISPOT assays were performed to quantify the number of total IgM and IgG1 ASCs in the spleen and BM and of autoantigen-specific IgM ASCs in the BM. 96-well ELISPOT plates (MSIPN4W50; MilliporeSigma) were prewetted with 50 μl of 35% ethanol, washed with PBS, coated with capture antibodies (3 μg/ml; 50 μl/well in PBS) and incubated at 4°C overnight. The plates were then washed with PBS and blocked with 200 μl culture medium for 2 h, and after additional washes, titrated numbers of splenocytes or BM cells were plated and incubated overnight at 37°C, 5% CO_2_. At the end point, cells were lysed (150 μl H_2_O/well; 10 min at room temperature) and plates were washed with 0.05% Tween-20 in PBS and incubated with HRP-conjugated antibodies for 2 h at 32°C. After additional washes (6× PBS–Tween-20; 2× H_2_O), plates were incubated with 3-amino-9-ethylcarbazole solution (551951; BD Biosciences) for 10 min to develop spots, washed extensively in H_2_O to stop reactions, and dried, and the spots were quantified using an ELISPOT reader (ImmunoSpot; CTL).

For total IgG1 and IgM assays, the following capture antibodies were used: goat anti-mouse IgM (RRID: AB_2794197, 1020-01; SouthernBiotech), goat anti-mouse IgG1 (RRID: AB_2794408, 1070-01; SouthernBiotech). We used goat anti-mouse Ig-HRP (RRID: AB_2728714, 1010-05; 1:5,000; SouthernBiotech) as a detection antibody.

For IgM autoantibody assay, the following mix of capture antigens was used: core histone (11010; Cayman Chemical Co.), ribophosphoprotein P2 (A14300; Surmodics), intrinsic factor (A16701; Surmodics), mitochondrial Ag (ATM02-10; Arotec Diagnostics), and SP100 (A18900; Surmodics; 3 μg/ml) in PBS and HRP-conjugated anti-mouse IgM (1:5,000) as a detection antibody. Splenocytes were titrated from 2 × 10^5^ cells/well and BM cells from 2 × 10^6^ cells/well. For BM B-1 and B-2 ELISPOT assays, BM cells were first enriched for CD19^+^ cells using CD19 microbeads (Miltenyi Biotec) and then sorted using a BD FACSAria II sorter by pregating on live, CD45^+^, GR-1^−^ and sorting CD19^+^B220^−^CD43^+^CD23^−^ (for B-1) and CD19^+^B220^+^ (for B-2) cells.

### Generation of BM chimeras

For generation of mixed BM chimeras, BM cells were isolated from WT and FL (*Foxp3*^Cre–ERT2^*CXCR4*^WT/WT^ or *Foxp3*^Cre–ERT2^*CXCR4*^FL/FL^, respectively) mice, which were maintained on a tamoxifen diet for 4–5 wk, and from CD45.1 mice (as described above). BM cells from WT and FL mice were T cell–depleted using Dynabeads FlowComp Mouse Pan T (CD90.2) Kit (Thermo Fisher Scientific), mixed with similarly prepared competitor (CD45.1) BM cells at a 1:1 ratio, and transplanted (1 × 10^6^ cells total) into lethally irradiated (950 rad) CD45.1 mice by i.v. injection.

### scRNA-seq analysis

Single-cell BM and spleen suspensions from two *Foxp3*^*DTR-EGFP*^ mice were pooled, and after CD4 T cell enrichment on magnetic beads, Treg cells (CD4^+^TCRβ^+^GFP^+^) and CD4conv T cells (CD4^+^TCRβ^+^GFP^−^) were FACS sorted, incubated with hash-tagged antibodies (BioLegend), diluted (1.5 × 10^3^ cells/μl in PBS, 0.04% BSA), pooled, and processed using Chromium X platform (10x Genomics). 50,000 and 44,432 CD4conv T cells and 46,230 and 20,827 Treg cells from spleen and BM, respectively, were used for these analyses.

#### scRNA-seq sample preprocessing

Individual samples were loaded on the 10x Genomics Chromium System. Libraries were prepared following 10x Genomics protocols (Chromium Single Cell 3′ Reagent Kits v2 Chemistry) and sequenced on NovaSeq 6000 (Illumina S2 flow cell, paired-end). FASTQ files were processed using Cell Ranger (http://support.10xgenomics.com/single-cell-gene-expression/software/pipelines/latest/what-is-cell-ranger), and reads were aligned to the mouse genome mm10 from ENSEMBL GRCm38. Cells that contained a percentage of mitochondrial transcripts >5% were filtered out, resulting in 2,159 splenic Treg cells and 1,887 BM Treg cells that passed quality control metrics. Cells with total molecule counts of <1,000, determined by lower mode of distribution of total molecules per cell, were additionally filtered out to remove putative empty droplets. Genes that were expressed in >10 cells were retained for further analysis. The resulting count matrices from all samples were then combined, resulting in a final set of 4,046 cells × 11,362 genes, and normalized to median library size, where library size is defined as total molecule counts per cell. The normalized data were then log transformed as log(counts +1) for downstream analysis.

#### Principle component analysis

For dimensionality reduction and visualization of data, genes with very low or very high expression of transcripts (log average expression <0.02 or >3 and dispersion >0.15) were excluded. Principal component analysis was performed on the log-transformed normalized data using 20 principal components, where the number of principal components was determined by the knee point in eigenvalues.

#### MAGIC imputation

To account for missing values in scRNA-seq due to the high dropout rate, imputation was performed using MAGIC (van Dijk et al., 2018). The diffusion maps matrix was constructed using *k* = 30, *ka* = 10, and *t* = 3 as input parameters, where *t* specifies the number of times the affinity matrix is powered for diffusion.

#### Clustering and gene ranking

Clustering of cells was performed using PhenoGraph (Levine et al., 2015) and setting *k* = 40 nearest neighbors. A cluster containing cells with a low number of total molecules compared with other populations was removed. Significant differentially expressed genes in each cluster were identified using *t* test implemented in Single-Cell Analysis in Python (SCANPY; Wolf et al., 2018) with default parameters. For identification of *Cxcr4*-expressing cells in scRNA-seq datasets, we used the MAGIC imputed matrix and set a cutoff of −3.05; any cell with a higher value was considered *Cxcr4* expressing (Cxcr4^+^; 446 cells). The rest of the population was considered *Cxcr4* nonexpressing (Cxcr4^−^).

### Colony-forming assay

BM colony-forming assay was performed by plating 10^4^ or 5 × 10^4^ cells on methylcellulose (MethoCult M3434; Stem Cell Technologies). Granulocyte/erythrocyte/macrophage/megakaryocyte, granulocyte/macrophage, granulocyte, macrophage, CFU-E (eythroid), and BFU-E (erythroid) colonies were counted 2 wk after seeding.

### Statistical analysis and data availability

Statistical analyses were performed using Prism software v8 (GraphPad). A statistical test was considered significant when P < 0.05. scRNA-seq data of spleen and BM CD4^+^ cells have been deposited to the Gene Expression Omnibus under accession no. GSM6135105.

### Online supplemental material

Fig. S1 shows general properties of CXCR4-expressing BM Treg cells. Fig. S2 shows the properties of thymic Treg cells and activation markers in FL mice. Fig. S3 shows the gating strategy of B-1 cells, properties of autoantibodies in FL mice, and results of other mouse models. Table S1 includes gene enrichment information for BM and spleen Treg cell clusters. Table S2 includes gene enrichment information of CXCR4^+^ BM and spleen Treg cell clusters.

## Supplementary Material

Table S1 is a cluster gene enrichment of combined BM and spleen Treg dataset: differential expression of all genes per cluster against the rest of clusters, where measures of score, fold change, and adjusted P value are reported. Score measurement is calculated by SCANPY algorithm and uses a formula based on fold changes and P values. The genes in each cluster are sorted based on their score.

Table S2 is a CXCR4^+^ gene enrichment of combined BM and spleen Treg dataset: list of all genes sorted by significance (SCANPY score) resulting from differential gene expression between CXCR4^+^ cells and the rest of cells in the dataset. Measurements of fold change and adjusted P value are also reported. CXCR4+ cells are identified as described in the Materials and methods.
